# Modeling Multispecies Gene Flow Dynamics Reveals the Unique Roles of Different Horizontal Gene Transfer Mechanisms

**DOI:** 10.3389/fmicb.2018.02978

**Published:** 2018-12-04

**Authors:** Phillip Nazarian, Frances Tran, James Q. Boedicker

**Affiliations:** ^1^Department of Physics and Astronomy, University of Southern California, Los Angeles, CA, United States; ^2^Department of Biological Sciences, University of Southern California, Los Angeles, CA, United States

**Keywords:** horizontal gene transfer, gene exchange simulation, extracellular vesicles, conjugation, transduction, transformation

## Abstract

Horizontal gene transfer within diverse bacterial populations occurs through multiple mechanisms of exchange. The most established routes of gene transfer, transduction, transformation, and conjugation, have been characterized in detail, revealing the advantages and limitations of each mechanism. More recently, interspecies gene exchange via extracellular vesicles has been reported and characterized, making vesicle-mediated exchange a fourth, general mechanism of gene transfer. Despite an understanding of each individual pathway, how all of these mechanisms act in concert has not been explored. Here we develop a model of gene exchange in a multispecies bacterial community that takes into account the rates and limitations of all four gene transfer mechanisms. Our results reveal unique roles for each gene exchange mechanism, and highlight how multiple pathways working together are required for widespread gene exchange within diverse bacterial populations.

## Introduction

Horizontal gene transfer (HGT) in bacteria is the exchange of genetic material between cells outside of reproduction. Because gene exchange by HGT occurs even between distantly related cells, HGT strongly influences bacterial evolution and enables bacterial populations to rapidly adapt to uncertainty in environmental conditions. Traditionally, three general mechanisms of HGT have been recognized and characterized in great detail: conjugation, transformation, and transduction (Griffith, 1928; Avery et al., 1944; Lederberg and Tatum, 1946; Zinder and Lederberg, 1952). Additional gene transfer agents have also been reported, which represent more specialized mechanisms of gene exchange that are not widely used by the majority of bacterial species (McDaniel et al., 2010; Lang et al., 2012). Recently, vesicle-mediated gene transfer has been identified as an additional HGT mechanism, and evidence is mounting that this method should be considered a fourth major route of gene exchange for bacteria (Yaron et al., 2000; Bushman, 2002; Renelli et al., 2004; Rumbo et al., 2011; Fulsundar et al., 2014; Tran and Boedicker, 2017).

Phylogenetic studies have repeatedly shown that HGT is prevalent and strongly influences bacterial evolution (Spratt et al., 2001; Nakamura et al., 2004; Pettersen et al., 2005). Estimates suggest 20% of bacterial genomes were acquired through horizontal transfer (Lawrence and Ochman, 1997). To explain this phenomenon, several models of HGT have been developed. Most models have investigated individual transfer mechanisms in isolation; others have focused on gene fixation only without regard to transfer mechanism (Levin et al., 1979; Nielsen and Townsend, 2004; Levin and Cornejo, 2009; Tayi et al., 2010; Lu et al., 2015; Niehus et al., 2015; Mao and Lu, 2016). No previous models have examined multiple HGT mechanisms acting simultaneously, and no models to date have incorporated vesicle-mediated HGT. Thus, despite a detailed, mechanistic understanding of HGT, there is a lack of quantitative understanding of how HGT occurs in real natural populations, where all of the mechanisms, each with unique advantages and limitations as detailed in Table 1, may be expected to influence overall dynamics and patterns of gene exchange. Here we develop such a comprehensive model of gene exchange within a model multispecies community of bacteria, revealing how each mechanism of HGT has a unique contribution to gene exchange dynamics.

**Table 1:** Four mechanisms of horizontal gene transfer.

Mechanism	Vector	Constraints	Rate factors
Conjugation	N/A: Direct cell-to-cell contact	Only possible for conjugative and mobilizable plasmids	Pairwise compatibility of donor and recipient
Transformation	Free extracellular DNA	Approximately **~**1% of species are naturally transformable (i.e., able to become competent and act as recipients)	Competence rate of recipient
Transduction	Transducing phages	Donor and recipient must both be in the host range of the transducing phage	Size of phage population common to donor and recipient
Vesicle-mediated transfer	Extracellular vesicles	Vesicle production and uptake rates low for some species	Efficiency of donor; efficiency of recipient

In this paper, we combine published information on the four major HGT mechanisms, including vesicle-mediated transfer, into one unified mathematical model. The model incorporates estimations of the rates and limitations of each of the four mechanisms. We then simulate a model community of 100 bacterial species to better understand how the properties of these four mechanisms interact to shape gene flow within diverse, multispecies microbial communities.

## Materials and Methods

This study uses an expanded version of the basic Levin mass-action model, which defines transfer rate as proportional to the product of donor and recipient concentrations (Levin et al., 1979). Over the past several decades, this model has been very widely and successfully used to study conjugation (Freter et al., 1983; Knudsen et al., 1988; Massoudieh et al., 2007, 2010; Wan et al., 2011; Zhong et al., 2012; Niehus et al., 2015). It has also been used previously to model transformation (Lu et al., 2015).

The following equation describes the gene transfer of a plasmid in a population consisting of *N* bacterial species:

(1) $$\begin{array}{c} \frac{dB_{i}^{+}}{dt}=\sum_{j=1}^{N}B_{i}^{-}(t)B_{i}^{+}(t)(\gamma_{conjugation\;}\;\alpha_{conjugation}(j,i)+\;\\ \gamma_{transformation}\alpha_{transformation}(j,i)\gamma_{transduction\;\;}\;\alpha_{transduction}(j,i)+\\ \gamma_{vesicle\;-\;mediated}\;\alpha_{vesicle\;-\;mediated}(j,i))\; \end{array}$$

*B_i_^+^(t)* is the concentration of bacterial species *i* containing the plasmid at time *t, B_i_^-^(t)* is the concentration of bacterial species *i* not containing the plasmid at time *t*, and γ is the optimal rate constant for each mechanism in units of mL cell^-1^ min^-1^. γ represents the rate of transfer between the fastest possible pair of donor and recipient species. The rate of transfer between any specific donor and recipient species may be less than that optimal rate (or even zero), depending on the rules of the mechanism. For each mechanism of transfer, pairwise rate modifiers between 0 and 1 are encoded in an *N*-by-*N* matrix *α* representing the compatibility of each donor with each potential recipient. For each mechanism, a single rate constant γ and a ruleset by which to create *α* compatibility matrices must be determined.

Unlike conjugation, which occurs through direct cell-to-cell contact, transformation, transduction, and vesicle-mediated transfer all involve some vector which transmits genetic material from donors to recipients (Bushman, 2002). For simplicity, the concentrations of these vectors are not directly modeled. Instead, any intermediate steps are accounted for in the rate constants and compatibility matrices for each mechanism, which describe transfer as one step from donor to recipient. This approach requires the reasonable assumption that the HGT vectors (i.e., free DNA, transducing phages, and extracellular vesicles) exist in the system at concentrations proportional to the concentrations of their source bacteria.

When implementing the model, several simplifications are made. First, the spatial distribution of the bacterial population is not considered. Spatial structure is known to have important consequences in gene exchange; here we model a well-mixed system, a reasonable approximation for aqueous environments (Sørensen et al., 2005; Zhong et al., 2012). Similar models have been used to explain conjugation measurements from spatially heterogeneous environments, suggesting that the model is in fact not totally inapplicable to non-aqueous bacterial populations (Knudsen et al., 1988; Massoudieh et al., 2007, 2010). A second key approximation is that the transferred plasmid is maintained within each recipient cell. We model a broad host range plasmid that can be replicated successfully within each species in the system. In reality, genetic elements have host ranges, and the model could be adapted to account for these differences, for example through modification of the compatibility matrix. A third key approximation is that cell growth is not explicitly considered. The total number of cells in the system does not change, nor do the relative population sizes of the species, which are assumed to be all constant and equal. The model simulates the spread of a genetic element, here a plasmid, through a population of cells that has reached a steady-state density and species composition.

### Fitness Parameter

We used 0.02 as an estimate for the average Malthusian fitness gain upon receiving the beneficial plasmid (Imhof and Schlotterer, 2001). This fitness parameter was used in all runs of our simulation. The equation used to describe the growth of the carrier of an advantageous plasmid within one species is:

(2) $$B_{t}^{+}=\frac{e^{\Delta t\;m}B_{t-1}^{+}}{1+\frac{B_{t-1}^{+}}{B_{t-1}^{+}+B_{t-1}^{-}}(e^{\Delta t\;\;m}-1)}$$

where *B_t_^+^* is the concentration of the cells containing the plasmid at timestep *t, B_t_^-^* is the concentration of the cells not containing the plasmid at timestep *t, m* is the Malthusian fitness parameter, and *Δt* is the length of the timestep. This equation follows directly from the mathematical definition of the Malthusian fitness parameter (Hartl and Clark, 2006). The fitness advantage of the plasmid is positive and of equal magnitude for all recipient species.

### Simulation

The mass-action model was implemented using MATLAB to simulate the spread of a plasmid within a community of *N* bacterial species interacting via HGT. For each run of the simulation, the timestep length, *Δt*, is 1/10000 of the simulation’s total length, which varies depending on the circumstances being simulated. The simulation is initialized with a starting state where only a single species contains the plasmid. At each time step, Equation 1 is first evaluated for every species to account for the contributions of the four HGT mechanisms. Second, the “vertical transfer” of the plasmid is evaluated according to Equation 2, which allows the plasmid to spread within each new species after horizontal transfer. For all simulations, a value of *N* = 100 was used, which is a reasonable estimate for the bacterial diversity of a sample of ocean water (Curtis et al., 2002; Torsvik et al., 2002).

It is important to note this method does not model individual transfer events, nor individual cells. Instead, the fraction of each species carrying the transferred plasmid is tracked over time. At early times the fraction of each species containing the plasmid is a very small number less than 1. The change of these values over time is controlled deterministically by Equation 1. Thus, there is no stochasticity in the simulation after the creation of the *α* matrices. This deterministic and non-discrete method of simulation is in keeping with the methodology and past uses of the Levin gene transfer model (Levin et al., 1979).

## Results

To implement the model described above, the maximum transfer rate, γ, and the exchange matrix, *α*, must first be established for each of the four mechanisms of transfer. These parameters were inferred from previously reported experimental measurements of HGT.

### Conjugation

The HGT mechanism of conjugation is facilitated by conjugative plasmids, which grant their hosts the ability to form cell-to-cell junctions. These junctions transfer the conjugative plasmid to the recipient cell via direct contact. Even if a plasmid is non-conjugative, it is still mobilizable via conjugation if it has an origin of transfer that allows it to “hitch a ride” on a conjugative pili created by another genetic element in the cell. Conjugation is not a viable mechanism for the horizontal transfer of non-conjugative, non-mobilizable plasmids (Bushman, 2002).

Since the Levin mass-action model has been widely used to study conjugation, there already exists an experimental method for measuring the rate constant γ of a donor-recipient pair. This so-called “end-point” method has been used to test a variety of donor-recipient pairs, and γ values between 10^-8^ and 10^-15^ mL cell^-1^ min^-1^ have been measured (Simonsen et al., 1990; Wan et al., 2011). Since our model defines γ as the optimal transfer rate, γ_conjugation_ is set to 10^-8^ mL cell^-1^ min^-1^.

The exchange matrix accounts for modulation of the maximum transfer rate between specific pairs of species due to potential barriers of gene exchange. This matrix specifies rules of exchange within a mixed population of microbes. Recent research has shown that certain broad host range plasmids are capable of conjugating into almost all species of bacteria (Klümper et al., 2015). However, certain pairs of bacteria conjugate more efficiently than others, due to a variety of still largely unknown factors. Relatedness between donor and recipient does not seem to have any consistent effect on transfer rate, even across taxa and the “Gram-barrier.” Additionally, approximately 15% of bacterial species—a “super-permissive core”—have been observed to uptake via conjugation at a rate approximately 25 times faster than the ordinary, non-super-permissive species. This super-permissive core is diverse and well-distributed across taxa (De Gelder et al., 2005; Klümper et al., 2015).

**FIGURE 1:**
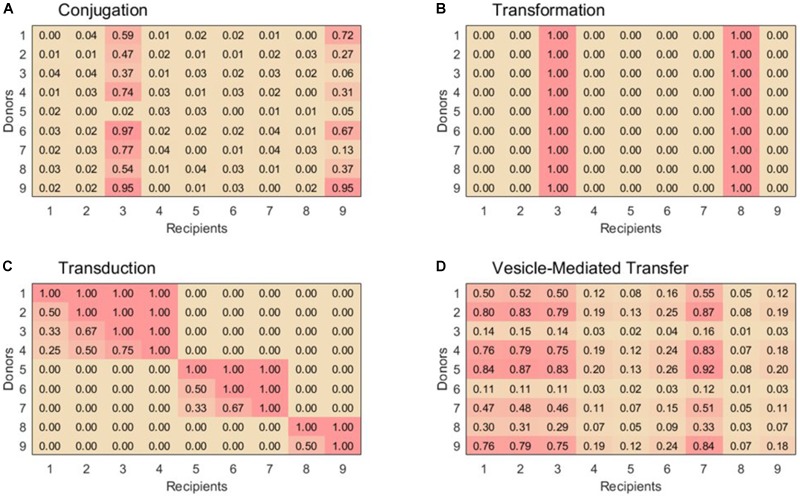
Example 9 × 9 compatibility matrices, α, for each horizontal gene transfer mechanism. **(A)** For conjugation, species 3 and 9 are part of the super-permissive core. **(B)** For transformation, only selected species can be recipient strains. Species 3 and 8 are naturally transformable whereas the other species are unable to uptake free DNA. **(C)** Transduction displays nested sets of species that are able to exchange phages. Within set of species that exchange phages, species with the highest exchange rates can be infected by all the phages in that module, while others with low exchange rates can only be infected by generalist phages. **(D)** Vesicle-mediated transfer has variable exchange rates, although as in conjugation all species are able to act as donors and recipients. Example matrices were chosen to emphasize key features for each transfer mechanism, see text for construction of matrices used in simulations.

For lack of better-defined patterns of conjugal interactions, our simulation uses a very simple ruleset to assign pairwise compatibilities. First, 15% of the recipient are randomly assigned to be in the super-permissive-core. For donor-recipient pairs containing a recipient in the super-permissive core, a random compatibility coefficient equally distributed between 0 and 1 is assigned. For donor-recipient pairs containing a recipient not in the super-permissive core, a random compatibility coefficient equally distributed between 0 and 1/25 is assigned to account for the lower rate of exchange. A sample exchange matrix for conjugation is shown in Figure 1A.

Although this scheme surely does not capture the full complexity of conjugal interactions, it is sufficient for representing the basic properties of conjugation in contrast to the other three HGT mechanisms (Figure 1).

### Calculating γ From Experimental Data for Transformation, Transduction, and Vesicle-Mediated Transfer

For the three HGT mechanisms besides conjugation, gene transfer experiments are typically performed by mixing recipient cells with vector, as opposed to donor cells. This vector is free DNA in the case of transformation, transducing phages in the case of transduction, and extracellular vesicles in the case of vesicle-mediated gene transfer (Ruhfel et al., 1984; Lu et al., 2015; Tran and Boedicker, 2017). Thus, the end-point method used to determine conjugation rate constants is not directly applicable. It is necessary to devise an alternative method by which to extract rate constants from published gene transfer data. In the mass-action formulation, the transfer dynamics of a typical vector-recipient gene transfer experiment are described by the following equations:

(3) $$\frac{dR}{dt}=-\gamma RV;\frac{dV}{dt}=-\gamma RV;\frac{dT}{dt}=\gamma RV$$

where *R* is the concentration of recipient cells, *T* is the concentration of transformed cells, and *V* is the concentration of the vector. All of these concentrations must be represented as quantity (or count) per volume, not mass per volume. By applying the same approach Simonsen et al. (1990) used to create the conjugation end-point formula, we used this set of equations to derive an “end-point” formula that can be applied to gene transfer experiments directly between a vector and recipients (see Supplementary Information for full derivation):

(4) $$\gamma =\frac{1}{\Delta t(V_{0}-R_{0})}(\ln(\frac{V_{0}-R_{0}+R_{2}}{R_{1}})-\ln(\frac{V_{0}}{R_{0}}))$$

*Δt* is the time interval of transfer, *V_0_* and *R_0_* are the initial concentrations of vector and recipient cells, respectively, and *R_1_* is the final concentration of recipient cells. All of these quantities are commonly measured and reported for gene transfer experiments (or at least easily derived from those that are). The γ in this formula defines the transfer rate per recipient cell concentration and vector concentration. To obtain a rate constant defined per recipient cell concentration and donor cell concentration, this γ must be scaled by a ratio of vector concentration to donor cell concentration.

Equations 3 and 4 are not directly part of our HGT model. Rather, these equations are only used to derive transfer rate constants from experimental data for transformation, transduction, and vesicle-mediated transfer.

### Transformation

Transformation is the uptake of free extracellular DNA from the environment by competent bacterial cells. The prevalence of transformation is limited by the fact that only a small number of bacterial species are “naturally transformable,” or capable of becoming competent (Bushman, 2002).

We used Equation 4 with data from a published transformation study to calculate our value of γ_transformation_ (Lu et al., 2015). This previous work used a mass-action model to model transformation measurements within a single species. With consideration of their success, we adopted their technique of using the mass of DNA and average DNA fragment size of 30 kb to convert between count and mass DNA concentrations. For example, a reported DNA concentration of 2.5 μg/mL at one data point translates to a *V_0_* of 8.33 × 10^11^ fragments/mL. Also accounted for is the assay’s use of recipient cells with artificially induced competence rates of approximately 0.2. Thus, for the same data point, the reported recipient concentration of 10^7^ cells/mL translates to an *R_0_* of 2 × 10^6^ cells/mL. Finally, the value of *R_1_* at that data point is easily calculated as 1.9998 × 10^6^ from the reported transformation frequency. From those three values, as well as a *Δt* of 30 min, Equation 4 yields γ = 4.00 × 10^-17^ mL DNA fragment^-1^ min^-1^. Repeating this calculation for each measurement reported in the paper generates a mean of γ = 4.35 × 10^-17^ mL DNA fragment^-1^ min^-1^.

To obtain our final γ_transformation_, this rate constant must be scaled to account for recipient competence rate and the ratio of free DNA to donor cells. In nature, the competence rates of bacterial species vary significantly; for this model, a reasonable competence rate of 0.01 is used (Lorenz and Wackernagel, 1994). γ_transformation_ is directly proportional to this competence rate; Supplementary Figure 1 shows that the choice of this parameter does not have a major impact on the outcome of the model. The ratio of vector concentration to donor cell concentration used is based on estimates of 10^6^ cells/mL and 0.01 μg/mL of DNA in ocean water, which translate to a ratio of 333 DNA fragments per donor cell given an average fragment size of 30 kb (Lorenz and Wackernagel, 1994; Jiang and Paul, 1998; Lu et al., 2015). With these two factors taken into account, we arrived at a final γ_transformation_ of approximately 10^-16^ mL cell^-1^ min^-1^.

Approximately 1% of bacterial species are naturally transformable. These species are well-distributed across taxa, so randomly choosing each species to be transformable with probability 0.01 is an appropriate rule to simulate natural transformation (Thomas and Nielsen, 2005; Johnston et al., 2014). The columns corresponding to these naturally transformable species are filled with the value 1 in the compatibility matrix; the others are filled by 0. A sample exchange matrix for transformation is shown in Figure 1B.

### Transduction

Transduction is the transfer of genetic material via transducing phages, which carry genetic cargo from donors to recipients. HGT via transduction is constrained by the host range of transducing phages. Transfer can only take place between donors and recipients that are both permissive to one or more of the same phages (Bushman, 2002).

Like transformation, transduction experiments are generally performed by mixing recipient cells directly with vector—in this case, transducing phages. Thus, Equation 4 can be used to calculate a rate constant for transduction.

We used data from a published transduction study to calculate our value of γ_transduction_ (Ruhfel et al., 1984). In this case, the initial concentration of transducing phages (tp) in the system was calculated from the reported concentration of recipient cells and multiplicity of infection (MOI). For example, reported values of *R_0_* = 6 × 10^8^ cells/mL and MOI = 0.4 in one experiment imply a *V_0_* of 2.4 × 10^8^ tp/mL. For that same experiment, the value of *R_1_* can be easily derived as 5.99999124 × 10^8^ cells/mL from the reported transductant concentration of 876 cells/mL. From those values, as well as a *Δt* of 60 min, Equation 4 yields γ = 1.01 × 10^-16^ mL tp^-1^ min^-1^. Repeating this calculation for the study’s other experiments reveals that this particular rate constant represents one of the most efficient donor-recipient pairs in the study. Thus, we used its value to calculate our final γ_transduction_, which is defined as the rate constant in the case of optimal donor-recipient compatibility.

To obtain γ_transduction_, this rate constant must be scaled by the ratio of transducing phages to donor cells. Estimates of 10^6^ cells/mL and 10^7^ phages/mL in ocean water imply a ratio of 10 phages per donor cell (Jiang and Paul, 1998). Thus, we arrived at a final γ_transduction_ of approximately 10^-15^ mL cell^-1^ min^-1^.

The ruleset for transduction is somewhat more complex than those of the other mechanisms. In their studies of phage host ranges, Weitz et al. (2013) have identified modularity and nesting as the two defining structures of phage-host interactions (Flores et al., 2011). In modular structures, distinct sets of phages infect distinct sets of hosts, with little to no overlap between these modules. In nested structures, generalist phages infect all hosts within a phage-host module and increasingly specialized phages infect smaller subsets of those hosts. In their analysis of phage-host interaction data sampled from natural ocean water, Flores et al. (2013) identified a multi-scale structure: on a large-scale, the interactions are modular, but within each module, nesting is apparent.

Thus, in order to create our compatibility matrix for transduction, we extrapolated the modularity and nesting structures of the phage-host networks into a host–host network. For simplicity, we defined both the size of our modules and the permissiveness of the hosts within each module to be distributed linearly. This estimation adequately matches the data reported by Flores et al. (2013) and also yields a distribution of phage population sizes consistent with that predicted by Breitbart et al. (2002) for ocean communities. With these structures established, the pairwise compatibility of a donor and a recipient is simply defined as the fraction of the donor’s hosted phages which can also infect the potential recipient. Donors and recipients in different modules are always assigned a compatibility of 0. A sample exchange matrix for transduction is shown in Figure 1C.

### Vesicle-Mediated Transfer

In vesicle-mediated gene transfer, recipients uptake extracellular vesicles that have been packed with genetic material and released by donors. Current research suggests that this route of transfer may allow for extremely broad transfer of plasmids without conjugation, since it lacks the constraints of transformation and transduction (Tran and Boedicker, 2017).

Like transformation and transduction, vesicle-mediated gene transfer experiments are performed by mixing recipient cells directly with vector—in this case, plasmid-loaded extracellular vesicles (EV). Thus, the same end-point formula we derived above can be used to calculate a rate constant for vesicle-mediated transfer.

We used data from a published vesicle-mediated gene transfer study to calculate our value of γ_vesicle-mediated_ (Tran and Boedicker, 2017). For example, reported values of *R_0_* = 4 × 10^9^ cells/mL, *V_0_* = 3.25 × 10^8^ EV/mL, and *R_1_* = 4.00 × 10^9^ – 1 cells/mL at one data point yield a rate constant of γ = 1.86 × 10^-21^ mL EV^-1^ min^-1^. Repeating this calculation for each of the paper’s data points generates a mean of γ = 4.76 × 10^-22^mL EV^-1^ min^-1^. The study’s estimate of 0.4 EV/cell was used as the ratio of vector concentration to donor cell concentration. Thus, we arrived at a final γ_vesicle-mediated_ of approximately 10^-22^ mL cell^-1^ min^-1^.

Vesicle-mediated gene transfer is not yet well enough studied to establish a population-level ruleset with much confidence. From what research has been reported, it seems that many bacterial species both produce and uptake extracellular vesicles loaded with genetic material. Different species display different efficiencies as donors and recipients, but pairwise compatibility factors are not known. The relatedness of the donor and recipient bacteria does not have any apparent correlation with the transfer rate, and there is no evidence that efficient recipients or donors are phylogenetically clustered (Tran and Boedicker, 2017). To model this behavior, each bacterial species is assigned two random values equally distributed between 0 and 1 to represent its donor and recipient efficiencies. In the vesicle-mediated transfer compatibility matrix, each donor-recipient pair is simply assigned the product of the applicable donor and recipient efficiencies. A sample exchange matrix for vesicle-mediated transfer is shown in Figure 1D.

### Rate Constants

**FIGURE 2:**
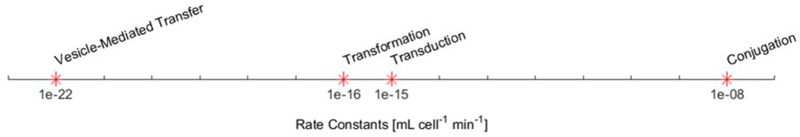
The derived maximum rate constants for the four HGT mechanisms.

As far as we are aware, there has never before been an attempt to quantitatively compare the rates of the four HGT mechanisms. The four rate constants derived here are shown in Figure 2.

These results are consistent with the common qualitative understanding of the HGT mechanisms. For example, it is commonly accepted that conjugation is the most prominent mechanism by a significant margin. It is to be expected that conjugation will dominate gene transfer for a conjugative plasmid. Transduction is usually considered to be a more important mechanism than transformation, which is to be expected since transformation is limited to only 1% of potential recipients (Bushman, 2002). Thus, the rate constant for transformation should not be interpreted as a direct measure of its relative importance in natural communities. Additionally, transduction’s rate constant is consistent with the findings of Volkova et al. (2014) who roughly estimated transduction to be 10^4^ times slower than conjugation in a cattle large intestine environment (Volkova et al., 2014).

As these rate estimates from the literature are approximate, additional analysis shown in Supplementary Figure 1 shows that adjusting any of these rate constants by as much as several orders of magnitude does not strongly change the conclusions drawn from simulation results.

### Transfer of a Conjugative Plasmid

To explore multispecies gene transfer dynamics, Equation 1 was simulated with the γ rate constants and *α* compatibility matrices for the four HGT mechanisms. When conjugation is turned on in the simulation (to simulate the transfer of a conjugative plasmid), conjugation dominates the other three HGT mechanisms. All bacterial species uptake the plasmid quickly, with the population achieving full spread (defined here as at least 99% plasmid uptake in every species) in a matter of days.

**FIGURE 3:**
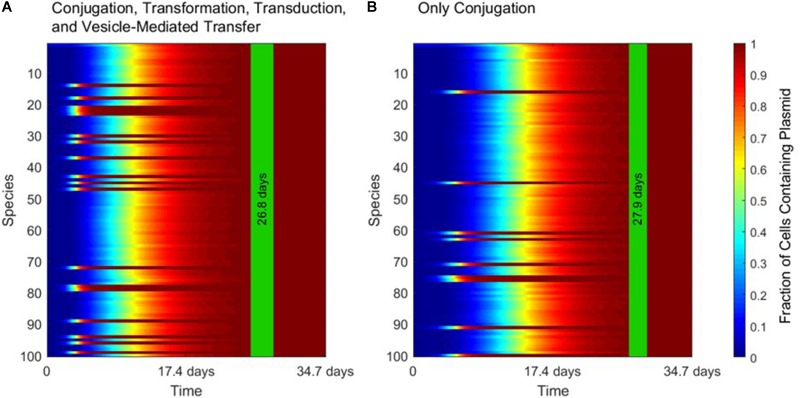
The strong influence of conjugation on horizontal gene transfer. In each panel, the vertical green stripe marks the mean time that the plasmid achieved full spread, plus and minus one standard deviation, for 5 repetitions of the simulation. Each plot shows uptake results for a single run. Full spread is defined as at least 99% plasmid uptake in every bacterial species. **(A)** In a simulation including all four gene transfer mechanisms, full spread occurred after 26.8 ± 1.4 days. **(B)** In a simulation with conjugation as the sole transfer mechanism, full spread occurred after 27.9 ± 1.1 days. The *p*-value of the time to full spread between the two experiments is 0.207 (two-tailed, unpaired *t*-test), which implies that in the presence of conjugation, the contribution of the other three mechanisms is not statistically significant.

Figure 3 demonstrates the dominance of conjugation as a gene transfer mechanism. As shown in Figure 3A, the transfer of a conjugative plasmid is very rapid, with full spread occurring in under 30 days. This result matches well with empirical results that, under selection pressure, conjugation can indeed spread a plasmid through a diverse population in a matter of days (Dionisio et al., 2002). The spikes in transfer for a few species correspond to members of the super-permissive core for conjugation. As will be explored further in the next section, non-conjugative, non-mobilizable plasmids took on average 80 times longer to spread through the population than conjugative plasmids in our simulation. As demonstrated by Figure 3B, none of the other three transfer mechanisms have a large impact on HGT dynamics when conjugation is active.

### Transfer of a Non-conjugative and Non-mobilizable Plasmid

**FIGURE 4:**
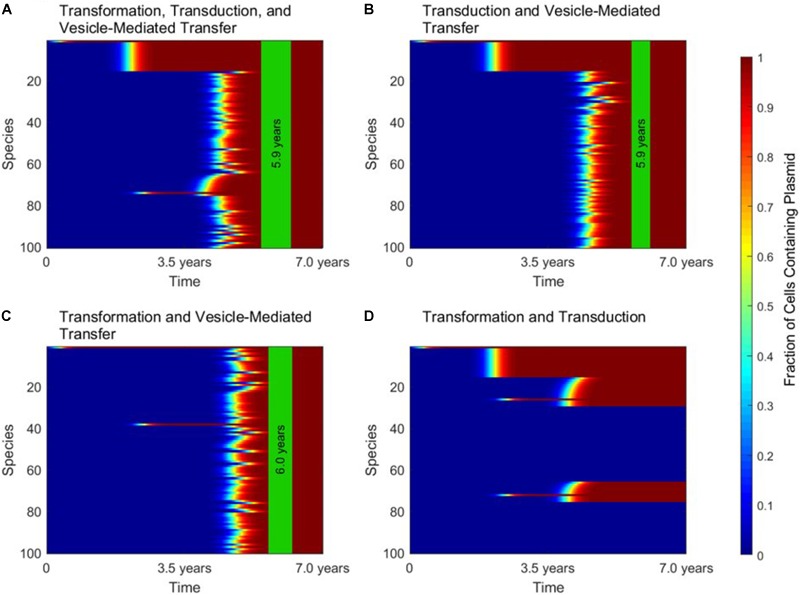
The roles of transformation, transduction, and vesicle-mediated transfer in horizontal gene transfer. For all four panels, conjugation did not occur, as in the case of a non-conjugative, non-mobilizable plasmid. In each panel, the vertical green stripe marks the mean time that the plasmid achieved full spread, plus and minus one standard deviation, for 5 repetitions of the simulation. Each plot shows uptake results for a single run. Full spread is defined as 99% plasmid uptake in every bacterial species. **(A)** For a simulation with transformation, transduction, and vesicle-mediated transfer, full spread occurred after 5.9 ± 0.4 years. **(B)** In a simulation without transformation, full spread occurred after 5.9 ± 0.2 years. **(C)** In a simulation without transduction, full spread occurred after 6.0 ± 0.3 years. **(D)** In a simulation without vesicle-mediated transfer, full spread was not achieved. The simulation terminated after 7.0 years, but full spread would never have occurred.

When conjugation is turned off in the simulation, to simulate the transfer of a non-conjugative, non-mobilizable plasmid, all three of the other mechanisms play important and unique roles. These roles are highlighted in Figure 4, in which gene transfer dynamics are compared when one of the three remaining gene transfer mechanisms was removed.

Figure 4A shows the dynamics that emerge when transformation, transduction, and vesicle-mediated transfer are all active. At early times, transformation serves the role of introducing the plasmid to any phage-host modules containing a naturally transformable species. Transduction then rapidly spreads the plasmid throughout the phage-host modules it enters. Vesicle-mediated transfer is the slowest of the three mechanisms, but it serves the critical role of introducing the plasmid to the phage-host modules that do not contain any naturally transformable species.

As shown in Figure 4B, without transformation, no phage-host modules uptake the plasmid at early times, so transduction only transfers the plasmid within the module in which the plasmid originates. However, the time to full spread is not significantly lengthened. As shown in Figure 4C, without transduction there is an initial transfer of the plasmid into species that participate in transformation and a lag before vesicle-mediated transfer spreads the plasmid throughout the entire population. The time to full spread is not significantly lengthened as compared to when transduction is included. As shown in Figure 4D, without vesicle-mediated transfer or conjugation, full spread never occurs. The plasmid is only able to enter phage-host modules containing a naturally transformable species and therefore is excluded for from several phage-host modules, regardless of the amount of time that passes. This result highlights the importance of vesicle-mediated transfer in the spread of non-conjugative, non-mobilizable plasmids.

**FIGURE 5:**
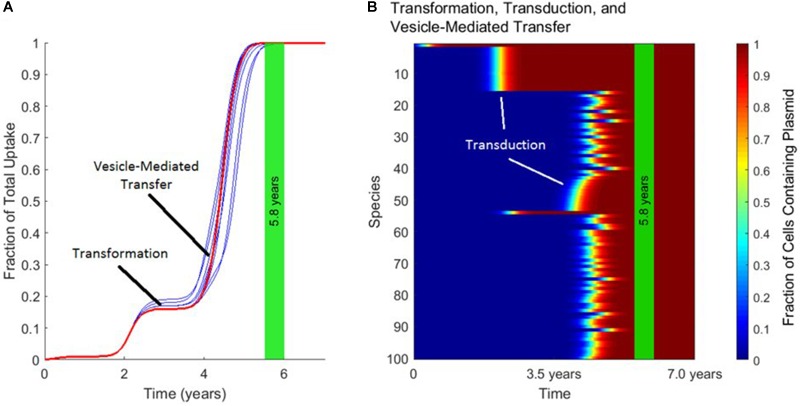
The timescales of transformation, transduction, and vesicle-mediated transfer. In each panel, the vertical green stripe marks the mean time that the plasmid achieved full spread, plus and minus one standard deviation, for 10 repetitions of the simulation. Full spread is defined as at least 99% plasmid uptake in every bacterial species. Full spread occurred after 5.8 ± 0.2 years. **(A)** The overall uptake of the plasmid over time for 10 runs of the simulation in the absence of conjugation. The approximate times at which transformation and vesicle-mediated transfer introduce the plasmid to new species are marked. Transduction continually spread the plasmid within each phage-host module throughout the simulation. **(B)** The per-species dynamics of the run marked in red in **(A)**. The first two phage-host modules in which the plasmid spread widely via transduction are marked.

Figure 5 demonstrates the distinct timescales on which transformation, transduction, and vesicle-mediated transfer influence gene transfer dynamics. As shown in Figure 5A, the uptake of the plasmid over time is characterized by a “stepping” pattern. The timing of these steps depends on the rate constants used for the three mechanisms. The jumps in fraction of total uptake occur at characteristic times set by the rate constant of each transfer mechanism. These characteristic times can also be observed in Figure 5B. The plasmid starts in Species 1, and around 2.0 years transduction spreads the plasmid to other species within the same phage-host module. Transfer also occurs via transformation around the same timescale, with transduction occurring again in this phage-host module around 4.0 years. Toward the end of the simulation, vesicle-mediated transfer further spreads the plasmid out amongst the remaining phage-host modules. In summary, transformation and vesicle-mediated transfer trigger the steps by introducing the plasmid into new phage-host modules. Transduction is ubiquitous throughout the gene transfer process and accounts for almost all of the transfer events occurring within each step.

## Discussion

We have created and simulated a mathematical model that relates all four known mechanisms of HGT. The results of the simulation provide new insight into the unique role that each mechanism serves in natural environments. For transfer of conjugative plasmids, conjugation is dominant because it has a broad transfer range and occurs at the fastest rate. For non-conjugative, non-mobilizable plasmids, the other three mechanisms each play an important and distinct role. Transformation is the second fastest mechanism, rapidly transferring the plasmid into a small number of naturally transformable species of bacteria. Transfer via transduction is apparent next, spreading the plasmid throughout bacterial phage-host modules. Vesicle-mediated transfer is the slowest HGT mechanism, but it plays the critical role of introducing non-conjugative, non-mobilizable plasmids into phage-host modules that do not contain any naturally transformable species. In the absence of conjugation and vesicle-mediated transfer, the plasmid did not spread to the majority of the species, highlighting the importance of gene transfer mechanisms with high promiscuity.

Vesicle-mediated transfer remains the least understood of the HGT mechanisms. Multiple species of both Gram-negative and Gram-positive bacteria have been shown to produce extracellular vesicles loaded with genetic material, and gene transfer between different species has been demonstrated (Jiang et al., 2014; Liao et al., 2014; Tran and Boedicker, 2017). Still, additional experimental studies are needed to determine if gene exchange via vesicles is as promiscuous as assumed in our calculations or if there are additional barriers to vesicle-mediated gene exchange between some species. Supplementary Figure 2 shows some simulation results where we model that not all species can produce and uptake vesicles. Previous measurements with a small set of species found no correlation between relatedness and transfer rate, but it is not clear if this pattern holds even for distantly related species. As in conjugation, vesicle-mediated transfer might also have a version of a “super-permissive core,” that is a set of species that exchange DNA in vesicles at a much higher rate.

Lack of consideration for spatially structured populations is an important shortcoming of our model. The different motilities and “lifespans” of the vector used by each gene transfer mechanism likely creates non-trivial gene transfer patterns over space. DNA within a vesicle or a phage capsid are protected from degradation, potentially enabling gene exchange over longer distances. Biofilms, a spatially complex arrangement of microbes, also have been identified as a hot-spot for HGT (Sørensen et al., 2005). Future work should attempt to augment our model with a technique for modeling the spatial distribution of cells. Some approaches to solving this problem have already been developed to model conjugation in isolation (Sørensen et al., 2005; Zhong et al., 2012).

Of course, there are many other factors influencing gene transfer dynamics that our model does not consider. Although one barrier to HGT is the physical process of moving a gene from a donor cell to the recipient cell, there are several additional barriers to HGT related to maintenance and fixation of the transferred gene in the recipient species. Microbes use restriction-modification systems and CRISPR interference to reduce the frequency of successful gene transfer events (Marraffini and Sontheimer, 2008; Bikard et al., 2012; Oliveira et al., 2016). Selection pressure is another barrier to fixation of a transferred gene (Thomas and Nielsen, 2005; Shapiro and Alm, 2008). Fixation of the transferred gene is an essential step in HGT, and has been the focus of several modeling efforts in HGT (Levin et al., 1979; Nielsen and Townsend, 2004; Levin and Cornejo, 2009; Tayi et al., 2010; Lu et al., 2015; Niehus et al., 2015; Mao and Lu, 2016). Relaxing the simplification in our model that the transferred gene experiences positive selection pressure in all species and is maintained within the recipient cell would impact HGT dynamics, both reducing gene transfer rates and preventing stable introduction of the gene into some species. Sequence divergence is another important factor for fixation involving homologous recombination (Majewski and Cohan, 1999), although non-homologous end joining can overcome these limitations and has been associated with increased HGT (Popa et al., 2011). Our model, which focuses on the initial barrier to HGT (moving a gene from a donor to recipient cell), does reveal the inter-mechanism dynamics of HGT despite removing the complexities of species and gene dependent fixation. Nonetheless, future work should attempt to combine models that incorporate the details of the gene delivery mechanisms with more realistic treatments of the gene in the recipient cell.

Though our model relies on many simplifications and approximations, it reveals how each of the HGT mechanisms could be expected to play a unique role in the gene transfer dynamics of complex, multispecies populations. Perhaps most interestingly, it shows that—despite being relatively slow—vesicle-mediated transfer is potentially a very important contributor to HGT. We hope that this result will motivate further research into the rate, promiscuity, and limitations of vesicle-mediated gene transfer. Also, given the unique advantages of transformation, transduction, and vesicle-mediated transfer revealed by these simulations, further experimental and theoretical exploration of the interplay of these gene transfer mechanisms acting within diverse microbial ecosystems is warranted. A deeper understanding of how multiple gene exchange mechanisms work together should result in a broader and more accurate picture of HGT in the wild. Different exchange mechanisms are likely responsible for the transfer of specific genes (including non-plasmid DNA) to specific donor-recipient pairs, potentially including gene transfer between bacteria and eukaryotic cells (Lacroix and Citovsky, 2016).

## Author Contributions

PN, FT, and JB designed the research, analyzed the data, and wrote the paper. PN conducted the research.

## Conflict of Interest Statement

The authors declare that the research was conducted in the absence of any commercial or financial relationships that could be construed as a potential conflict of interest.
