# Supplementary material for: Modeling Multispecies Gene Flow Dynamics Reveals the Unique Roles of Different Horizontal Gene Transfer Mechanisms
Source: Front Microbiol. 2018 Dec 4;9:2978. doi: 10.3389/fmicb.2018.02978 (PMC6288448; doi:10.3389/fmicb.2018.02978)
Supplement: Supplementary file 1 [file Data_Sheet_1.PDF]

Supplementary Information for:

Modeling multispecies gene flow dynamics reveals the unique roles of different horizontal gene transfer mechanisms.

Phillip Nazarian, Frances Tran and James Q. Boedicker

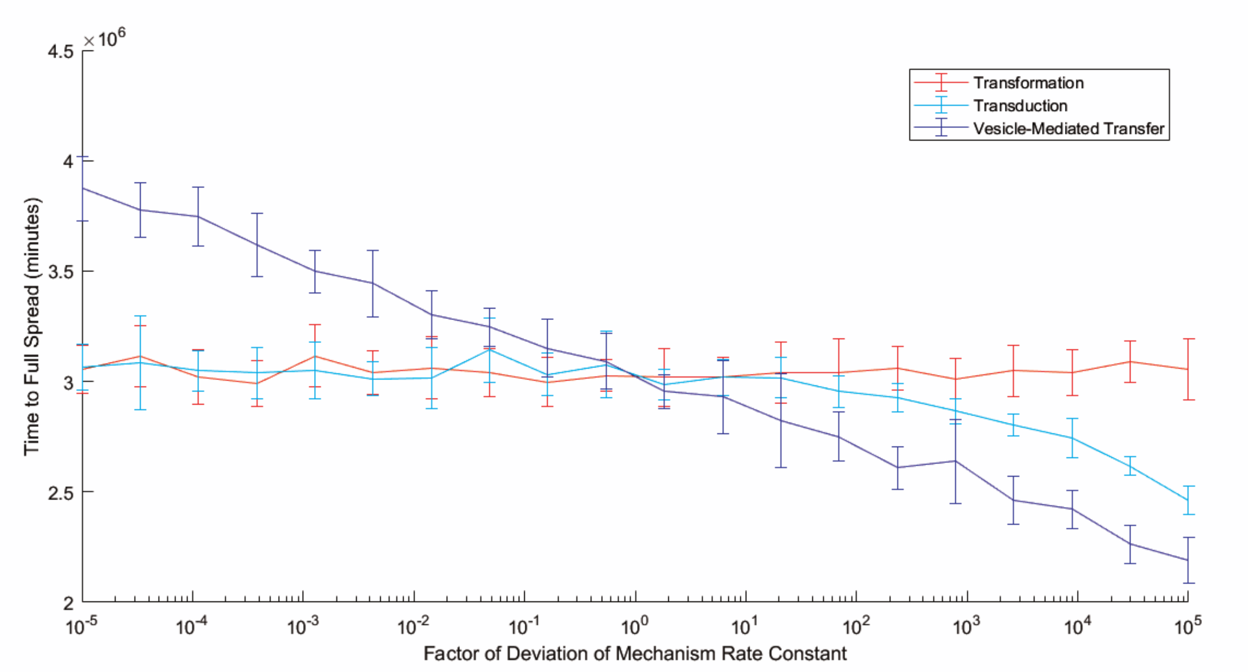

SI Figure 1.

Sensitivity of overall gene transfer dynamics to the rate constants when conjugation does not occur, as in the case of a non-conjugative and non-mobilizable plasmid. Along the x-axis, the rate constant for one HGT mechanism at a time is varied by a factor ranging from 10^{-5} to 10^{5}. The y-axis plots the time to full spread, defined as 99% plasmid uptake in every bacterial species. Error bars show standard deviation for 10 runs of the simulation at each data point. Varying the rate of transformation has no significant effect on the time to full spread. The rate of vesicle-mediated transfer demonstrates a nearly perfect logarithmic relationship with the time to full spread. Reducing the rate of transduction has no significant effect on the time to full spread, but increasing it by a few orders of magnitude significantly reduces the time to full spread.

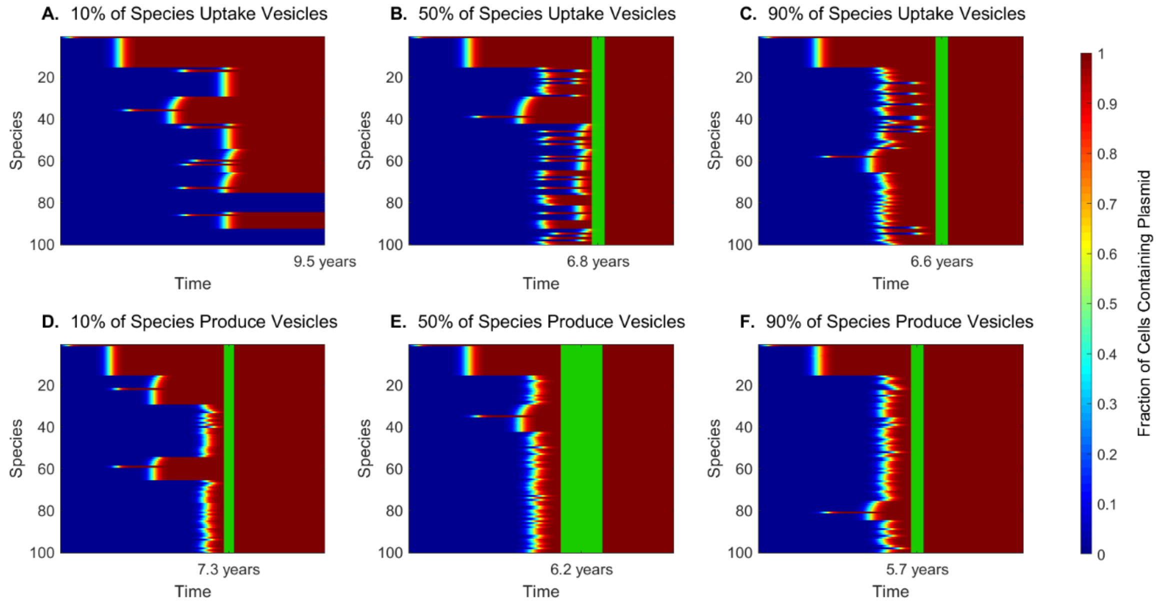

SI Figure 2.

Gene transfer dynamics with alternative rules for vesicle-mediated exchange. In these simulations, conjugation did not occur, as in the case of a non-conjugative, non-mobilizable plasmid. In each panel, the vertical green stripe marks the mean time that the plasmid achieved full spread, plus or minus one standard deviation, for 5 repetitions of the simulation. Each plot shows uptake results for a single run. Full spread is defined as 99% plasmid uptake in every bacterial species. (A) In a simulation where only 10% of species uptake vesicles, full spread was not achieved. The simulation terminated after 9.5 years, but full spread would never have occurred. (B) In a simulation where only 50% of species uptake vesicles, full spread occurred after 6.8 years, on average. (C) In a simulation where only 90% of species uptake vesicles, full spread occurred after 6.6 years, on average. (D) In a simulation where only 10% of species produce vesicles, full spread occurred after 7.3 years, on average. (E) In a simulation where only 50% of species produce vesicles, full spread occurred after 6.2 years, on average. (F) In a simulation where only 90% of species produce vesicles, full spread occurred after 5.7 years, on average.

Derivation of Equation 4, the end-point formula for vector-mediated horizontal gene transfer:

1	$\frac{dR}{dt} = -\gamma RV, \quad \frac{dV}{dt} = -\gamma RV, \quad \frac{dT}{dt} = \gamma RV$	Equation 3, the dynamics of the system being modeled.
2	$Q = \frac{V}{R}$	Define a new variable Q to help with the derivation.
3	$\frac{dQ}{dt} = \frac{d}{dt}\left(\frac{V}{R}\right) = \frac{R\frac{dV}{dt} - V\frac{dR}{dt}}{R^2}$	Differentiate Q with respect to time.
4	$\frac{dQ}{dt} = \frac{-\gamma R^2 V + \gamma R V^2}{R^2} = \gamma Q(V - R)$	Substitute in from step 1 and simplify based on the definition of Q (step 2).
5	$\gamma = \frac{1}{V_o - R_o}\left(\frac{\frac{dQ}{dt}}{Q}\right)$	Solve for gamma. Notice that (V-R) is constant with respect to time, so its value at any time can be used.
6	$\int_{t_0}^{t_1} \gamma\, dt = \frac{1}{V_o - R_o}\int_{t_0}^{t_1} \frac{\frac{dQ}{dt}}{Q}\, dt = \frac{1}{V_o - R_o}\int_{t_0}^{t_1} \frac{1}{Q}\, dt$	Integrate over time.
7	$\gamma(t_1 - t_0) = \frac{1}{V_o - R_o}(\ln(Q_1) - \ln(Q_0))$	Solve the integral.
8	$\gamma = \frac{1}{\Delta t(V_o - R_o)}(\ln(Q_1) - \ln(Q_0)) = \frac{1}{\Delta t(V_o - R_o)}\left(\ln\left(\frac{V_1}{R_1}\right) - \ln\left(\frac{V_0}{R_0}\right)\right)$	Solve for gamma and substitute back the definition of Q from step 2.
9	$\gamma = \frac{1}{\Delta t(V_o - R_o)}\left(\ln\left(\frac{V_0 - R_0 + R_1}{R_1}\right) - \ln\left(\frac{V_0}{R_0}\right)\right)$	Notice that $V_1 = V_0 - (R_0 - R_1)$.

Codes for simulation of horizontal gene transfer. Written for Matlab R0217a.

Attached as separate files are:

hgt.m – the core code that simulates hgt via the four mechanisms of exchange
constants.m – defines constants used for HGT simulations
format_time_interval.m – adjusts the unit of time reported for simulations

In addition, files such as figure3.m are included. To re-simulate data reported in Figure 3, transfer hgt.m, constants.m, and format_time_interval.m to your active folder in Matlab and run figure3.m.
